# Type I error control and interim monitoring for co-primary hypotheses involving a subgroup in the Outpatient Treatment with Anti-Coronavirus Immunoglobulin (OTAC) trial

**DOI:** 10.1016/j.conctc.2025.101592

**Published:** 2025-12-24

**Authors:** Jiayi Hu, Abdel G. Babiker, Cavan S. Reilly, Jason V. Baker, Lianne K. Siegel

**Affiliations:** a Division of Biostatistics and Health Data Science, School of Public Health, University of Minnesota, MN, USA; b Medical Research Council Clinical Trials Unit at UCL, University College London, London, UK; c Division of Infectious Diseases and International Medicine, University of Minnesota, MN, USA; d Division of Infectious Diseases, Hennepin Healthcare, MN, USA

**Keywords:** Clinical trials, Multiple testing, Nested subgroups, Type I error rate, Group sequential design

## Abstract

The recent growth of immunoglobulin-based therapies has motivated clinical trials testing primary endpoints both in the overall cohort and in subgroups of patients, such as in patients without specific antibodies at baseline. Multiple testing methods in clinical trials often ignore the natural correlation between test statistics in such contexts, resulting in overly conservative type I error control. The Outpatient Treatment with Anti-Coronavirus Immunoglobulin (OTAC) trial, is an ongoing Phase III trial evaluating the effect of a single infusion of anti-COVID-19 hyperimmune intravenous immunoglobulin (hIVIG), in outpatient adults with recently diagnosed SARS-CoV-2 infection, in both the overall cohort and in the subgroup of participants who had not received monoclonal antibodies or antiviral treatments. We present the method used to control the type I error at a predetermined rate while taking the estimated correlation into account, thus increasing efficiency. We evaluated the operating characteristics of this method in both fixed and group-sequential scenarios through extensive simulation studies. Our findings indicate that this approach controls the type I error at the desired rate, improves power, and reduces the expected sample size compared to a Bonferroni correction. **Trial Registration**: This study was registered on clinicaltrials.gov under NCT0491026 on 1 June 2021.

## 1 Introduction

This article describes the statistical approach of controlling the type I error rate in the Outpatient Treatment with Anti-Coronavirus Immunoglobulin (OTAC) trial, testing the treatment effect of anti-COVID-19 hyperimmune intravenous immunoglobulin (hIVIG) in both the overall population and a subgroup of the population in the primary analysis. Specifically, we consider two co-primary hypotheses comparing hIVIG versus placebo in the overall outpatient population and a nested subgroup expected to have a larger treatment effect. The practical challenge is to control the family-wise type I error rate for these correlated tests, while allowing for interim monitoring.

Immunoglobulin-based therapy includes the use of monoclonal antibodies, which were widely used during the COVID-19 pandemic, [1], [2], [3], [4], [5], [6], [7], [8], immunoglobulin replacement therapy [1], [9], [10], and immune checkpoint inhibitors [11], [12], among other treatments; this has become a common treatment in infectious diseases [1], [3], [4], auto-immune diseases [5], [12], transplantation [6], [7], [8], and several rheumatic diseases [2], [10], and its applications are still being explored [13], [14]. In the context of immunoglobulin-based therapy, evidence suggests that the treatment effect may be more pronounced among participants who lack pre-existing antibodies [15], [16] or do not use other antivirals. Therefore, clinical trial designs have prioritized testing the treatment effect in such subgroups in addition to the entire cohort.

The OTAC trial (clinicaltrials.gov identifier: NCT04910269), a phase III, multi-center, double-blind, randomized, placebo-controlled trial, is testing the safety and efficacy of a single infusion of anti-coronavirus hIVIG versus placebo among adult outpatients with recently diagnosed coronavirus 2 (SARS-CoV-2). The trial has two strata, distinguished by whether participants are receiving or plan to receive direct-acting antivirals (DAAs) or other available anti-SARS-CoV-2 agents recommended for use as part of standard of care (SOC). Stratum 1 includes participants not planning to receive these agents, and is hypothesized to have a larger treatment effect compared to stratum 2 due to the absence of other antiviral interventions. The primary efficacy outcome will be evaluated both in the overall population and specifically within stratum 1.

This leads to the need to control type I error in the presence of multiple correlated tests, as stratum 1 is a subset of the overall population. The TICO and RECOVERY studies provide two high-profile examples of other monoclonal antibody trials testing co-primary hypotheses of the treatment effect in the full cohort and in a subgroup. The tixagevimab–cilgavimab trial under the TICO master protocol [1], [17], [18], [19], [20], tested the treatment effect in the full cohort and in patients seronegative for anti-SARS-CoV-2 neutralizing antibodies at baseline using Holm’s method [21]. The lower of the two p-values was first compared to 0.025, corresponding to a Bonferroni correction. If significant, then the other p-value was compared to 0.05. Here, power is limited by the threshold of 0.025 for the lower p-value, which does not incorporate the correlation between tests. The RECOVERY trial [22] used a fixed-sequence method [23] to test the monoclonal antibody combination of casirivimab and imdevimab on hospitalized patients with COVID-19 in both the overall population and also in baseline seronegative subgroup. The treatment was first tested among seronegative participants, and if significant at the 0.05-level, tested in the overall cohort, requiring this order of tests to be pre-specified. Other methods have also been used for controlling the type I error rate in clinical trials with multiple tests, including the Bonferroni correction [21], Benjamini–Hochberg procedure [24], prospective alpha allocation scheme (PAAS) [25], and gate-keeping strategy [26]. However, none of the methods above leverage the correlation between tests.

Several methods have been proposed to address multiple testing in clinical trials when accounting for correlated tests. Dunnett’s test [27] compares multiple groups against the same control while accounting for the correlation between tests. Bretz and Maurer proposed a graphical approach for lowering the nominal p-value based on the correlation between tests [28] including within group sequential designs [29]; its extension [30] allows for the consideration of correlations between outcomes in a fixed design; Anderson et al. [31], [32] extend the framework further to a group sequential design. However, this work primarily focuses on theoretical frameworks and does not directly address that the correlation between tests is unknown at the outset of the trial and must be estimated using the observed data; this is counter-intuitive as the nominal p-value must be determined by the observed data and presents specific challenges for interim monitoring. Joo et al. [33], who illustrate the use of prospective alpha allocation in the context of the COAG trial where the correlation is unknown, incorporate the correlation between tests but do not consider interim monitoring. Sinha et al. [34] developed an adaptive enrichment group sequential design for two binary co-primary endpoints, where the subgroups are disjoint. Chen et al. [35] introduced a complete-correlation framework for group sequential trials with nested biomarker subpopulations and temporal consideration, but their method calculated the alpha spending over time at the trial design stage and did not consider recalibrating the nominal critical values when the actual correlations differed from those originally assumed.

Bayesian approaches to multiple testing and subgroup evaluation can also account for dependence across hypotheses through hierarchical modeling and decision-theoretic rules [36], [37], [38], but these methods do not typically provide the strict control of the type I error rate required for confirmatory regulatory trials such as OTAC.

In this case study, we describe the statistical details of controlling type I error in the OTAC trial by lowering the nominal p-value used for each test based on the estimated correlation, which has a closed form. We simulate the operating characteristics under scenarios where the correlation between tests is estimated from the observed data at the end of the trial, with particular consideration given to interim monitoring and the consequences of the correlation differing from value assumed at the design stage. Section 2 describes the sample size assumptions used when designing the OTAC trial. We describe the methods for controlling type I error in Section 3 and illustrate the application in Section 4. Simulation methods, results with and without interim analyses are presented in Section 5. A discussion of the simulation findings, limitations, and future directions is given in Section 6.

## 2 Motivating example - OTAC

The primary outcome of the OTAC trial is a 5-category ordinal outcome assessing the participant’s clinical status 7 days after the infusion of hIVIG or placebo, with a value of 5 being the most severe, and 1 being the least severe. The treatment effect of hIVIG is evaluated both for all randomized participants and for only those in stratum 1.

Several key assumptions were made at the beginning of the OTAC trial when determining the sample size:

• 1:1 allocation ratio of treatment to control in both strata.
• 80% of participants enrolled in stratum 1, and the proportions of participants in each ordinal outcome category (from 1 to 5) in the stratum 1 placebo group are assumed to be (0.2,0.4,0.25,0.12,0.03).
• An odds ratio (OR) greater than 1 indicates “better” outcomes. The placebo group in stratum 2 has a common OR of 1.72 relative to the placebo group in stratum 1. Under the alternative hypotheses, the summary ORs comparing treatment against placebo are 1.5 for stratum 1 and 1.2 for stratum 2.

## 3 Methods

### 3.1 Type I error rate control with a single analysis

#### 3.1.1 Correlation between maximum likelihood estimates

Let θ denote the treatment effect in the overall population, and $\theta_{i}$ denote the treatment effect in stratum i, i=1,…,n. Then let U and $U_{i}$ denote the asymptotically unbiased maximum likelihood estimators for θ and $\theta_{i}$, and let v and $v_{i}$ denote the corresponding variances of U and $U_{i}$. Thus, the $U_{i}$s are independent and normally distributed with $E\left(U_{i}\right)=\theta_{i}$ and $Var\left(U_{i}\right)=v_{i}$ for i=1,…,n. U is asymptotically the weighted average of the $U_{i}$s, with weights proportional to the precision of each estimator [39]. It can also be shown that this applies to partial likelihood estimates as well.

(1) $$U=\underset{i}{\sum }w_{i}U_{i},\;\;\text{where}\;\;w_{i}=\left(1/v_{i}\right)/\underset{j}{\sum }1/v_{j},\;i=1,\ldots ,n.$$

Thus, the covariance between U and $U_{i}$ is as follows:

(2) $$cov\left(U,U_{i}\right)=cov\left(\underset{j}{\sum }w_{j}U_{j},U_{i}\right)$$

(3) $$=cov\left(w_{i}U_{i},U_{i}\right)=w_{i}v_{i},\;i=1,\ldots ,n,$$

and the correlation between U and $U_{i}$ is then given by:

(4) $$corr\left(U,U_{i}\right)=\frac{cov\left(U,U_{i}\right)}{\sqrt{vv_{i}}}=\frac{w_{i}v_{i}}{\sqrt{vv_{i}}}=\sqrt{\frac{w_{i}^{2}v_{i}}{v}}=\sqrt{w_{i}^{2}v_{i}/\underset{j}{\sum }w_{j}^{2}v_{j}}=\sqrt{1/\left(v_{i}\underset{j}{\sum }1/v_{j}\right)},\;i=1,\ldots ,n.$$

#### 3.1.2 Nominal P-value

Let $H_{0}$ and $H_{0,i}$ denote the null hypotheses that the treatment effect equals 0 overall and in stratum i, respectively, i=1,…,k. Here, k≤n denotes the number of strata included in the co-primary testing set. In the OTAC trial, for example, we pre-specified n=2 strata and included k=1 strata (Stratum 1) in the co-primary hypothesis. Let α be the type I error rate; we assume that type I error is spent equally between tests, and thus the nominal p-values for all tests will be the same, denoted by $p^{*}$. The critical values are denoted by c for all tests, where $c=\Phi^{-1}\left(1-\frac{p^{*}}{2}\right)$. Z denotes the observed Z-score for the test for the overall population, $Z_{i}$ for the test in the ith stratum, where $Z=\left|U\right|/\sqrt{v}$ and $Z_{i}=\left|U_{i}\right|/\sqrt{v_{i}}$. The following calculation is based on a two-sided test, and the corresponding nominal p-values for a one-sided test can be obtained by doubling the nominal p-value obtained for a two-sided test so that the critical value becomes $\Phi^{-1}\left(1-p^{*}\right)$.

(5) $$P\left(\left|Z\right|>c|H_{0}\right)=P\left(\left|Z_{i}\right|>c|H_{0,i}\right)=p^{*},\text{for}i=1,\ldots ,k.$$

Thus, we want an appropriate $p^{*}$ to control the overall type I error rate at a pre-specified α level (e.g. 0.05). Under the null hypothesis, $\left(U,U_{1},\ldots ,U_{k}\right)$ follows a multivariate normal distribution with the mean equal to (0, …,0) and variance–covariance matrix Σ as follows:

$$\Sigma =\left(\begin{array}{cccc} v & \text{corr}\left(U,U_{1}\right)\cdot \sqrt{vv_{1}} & \cdots & \text{corr}\left(U,U_{k}\right)\cdot \sqrt{vv_{k}}\\ \text{corr}\left(U,U_{1}\right)\cdot \sqrt{vv_{1}} & v_{1} & 0 & \cdots \\ \vdots & 0 & \ddots & \ddots \\ \text{corr}\left(U,U_{k}\right)\cdot \sqrt{vv_{k}} & \vdots & \ddots & v_{k} \end{array}\right).$$

The nominal p-value $p^{*}$ is chosen so that the family-wise type I error equals α, i.e., $p^{*}$ is the largest value satisfying

(6) $$\;\;P\left(\left(\left|U\right|>c\sqrt{v}\right)\cup \left(\left|U_{1}\right|>c\sqrt{v_{1}}\right)\cup \cdots \cup \left(\left|U_{k}\right|>c\sqrt{v_{k}}\right)|H_{0},H_{0,1},\ldots ,H_{0,k}\right)=1-P(\left(\left|U\right|\leq c\sqrt{v}\right)\cap \left(\left|U_{1}\right|\leq c\sqrt{v_{1}}\right)\cap ...\cap \left(\left|U_{k}\right|\leq c\sqrt{v_{k}}\right)|$$

$$\;\;\;\;\;\;\;\;\;\;\;\;\;H_{0},H_{0,1},\ldots ,H_{0,k})=\alpha .$$

This probability can be evaluated by integrating the (k+1)-dimensional multivariate normal distribution with the following limits

$$\left(-c\sqrt{v},\;-c\sqrt{v_{1}},\;\ldots ,\;-c\sqrt{v_{k}}\right)\;\text{and}\;\left(c\sqrt{v},\;c\sqrt{v_{1}},\;\ldots ,\;c\sqrt{v_{k}}\right).$$

Because Eq. (6) does not admit a closed-form solution for the two-sided nominal level $p^{*}$, we determine $p^{*}$ numerically. For example, with two co-primary tests, as in the OTAC trial, we search over candidate values $p^{*}\in \left[0.025,0.05\right]$ on a fine grid (step size 0.0001). For each candidate $p^{*}$, we set $c=\Phi^{-1}\left(1-p^{*}/2\right)$ and compute the joint probability of rejecting at least one null hypothesis under $\left(H_{0},H_{0,1}\right)$ using the corresponding bivariate normal distribution. We then choose the largest $p^{*}$ such that this joint probability does not exceed the prespecified family-wise type I error α. In practice, the true standard deviations are unknown, especially at the beginning of the trial; thus, we use the observed standard errors of the treatment effect estimates at the end of the trial to determine $p^{*}$.

### 3.2 Interim monitoring

Suppose for each hypothesis test, we have M planned analyses (i.e. M−1 interim analyses); the $p^{*}$ is first designated at the beginning of the trial to be the theoretical type I error rate for each hypothesis test obtained from the assumptions made during the trial design. At analysis m=1,…,M, let $t_{m}$ denote the information fraction; $p_{m}$ denote the corresponding amount of type I error spent, i.e. $\sum_{m=1}^{M}p_{m}=p^{*}$; $c_{m}$ denote the critical value; $Z_{i}\left(t_{m}\right)$ denote the observed interim z-score.

Suppose we want to adjust the critical value based on the current data in the $m^{th}$ analysis time, m=1,…,M. If m=1, the new $p^{*}$ will be calculated from the observed standard errors for the test statistics, and the amount of type I error to spend by time $t_{1}$ will be consequently obtained using a pre-specified alpha spending function. If m>1, the updated critical value $c_{m}$ should be adjusted to satisfy:

(7) $$P\left(Z_{i}\left(t_{1}\right)>c_{1}\right)+P\left(\left(Z_{i}\left(t_{1}\right)\leq c_{1}\right)\cap \left(Z_{i}\left(t_{2}\right)>c_{2}\right)\right)+\cdots +\;P\left(\left(Z_{i}\left(t_{1}\right)\leq c_{1}\right)\cap \cdots \cap \left(Z_{i}\left(t_{m-1}\right)\leq c_{m-1}\right)\cap \left(Z_{i}\left(t_{m}\right)>c_{m}\right)\right)=p_{new}^{*},$$

where $p_{new}^{*}$ is calculated based on the *observed* data at $m^{th}$ time point from Eq. (6). Given a pre-specified spending function $\alpha_{*}\left(t\right)$, a new spending function $\alpha_{*}^{\prime }\left(t\right)$ based on $p_{new}^{*}$ can be derived according to [40]:

(8) $$\frac{\alpha_{*}\left(t\right)-\alpha_{*}\left(t_{m}\right)}{p^{*}-\alpha_{*}\left(t_{m}\right)}=\frac{\alpha_{*}^{\prime }\left(t\right)-\alpha_{*}\left(t_{m}\right)}{p_{new}^{*}-\alpha_{*}\left(t_{m}\right)}$$

$$\alpha_{*}^{\prime }\left(t\right)=\frac{\left(p_{new}^{*}-\alpha_{*}\left(t_{m}\right)\right)\left(\alpha_{*}\left(t\right)-\alpha_{*}\left(t_{m}\right)\right)}{p^{*}-\alpha_{*}\left(t_{m}\right)}+\alpha_{*}\left(t_{m}\right),$$

where $t_{m}$ is the information time at the previous look.

**Fig. 1:**
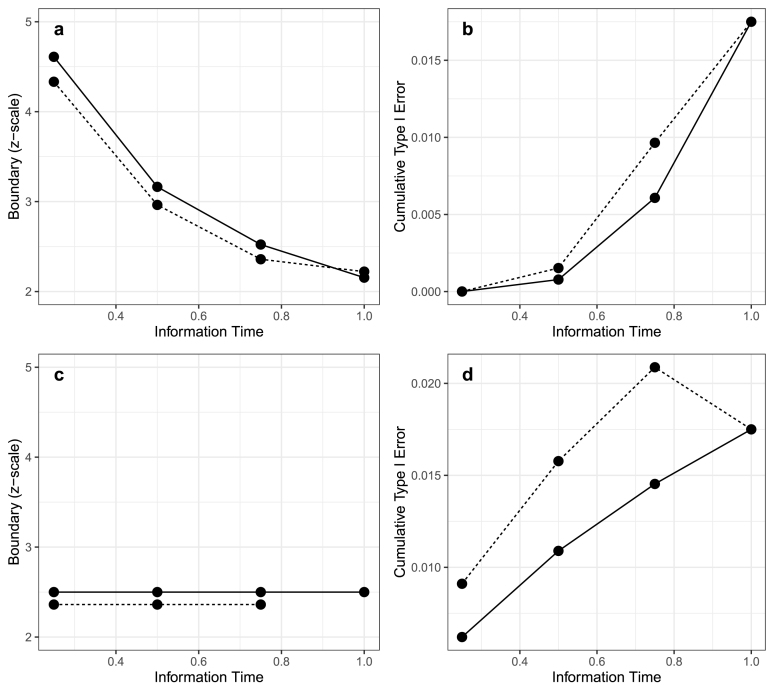
Interim monitoring boundaries and cumulative alpha spending correcting at each analysis (solid line) versus correcting only at the final analysis (dashed line) for O’Brien Fleming-like alpha spending function (a–b) and Pocock-type alpha spending function (c–d) for a two strata design.

The $p^{*}$ based on the full trial data will be no smaller than 0.05 divided by the number of tests k+1, which corresponds to a Bonferroni correction assuming independence between the tests; thus, as long as no more than 0.05/(k+1) in type I error is spent in the first M−1 interim analyses, the alpha spending function for the co-primary tests based on the estimated correlation can be adjusted only at the final look without spending more than the pre-specified type I error. In the context of a Lan-DeMets O’Brien Fleming-like spending boundary, a small portion of the type I error is allocated to the first M−1 interim analyses, conserving most type I error for the final analysis and maintaining the power of the hypothesis test. In this case, the benefit of adjusting the alpha spending functions before the final analysis is uncertain due to minor differences in the earlier ones. Fig. 1 panels a and b illustrate this point by showing an example of interim stopping boundaries on the z-scale and the cumulative alpha spent when adjusting the alpha spending function during the trial compared to only at the end. We assumed that there were two hypothesis tests and three interim analyses at $t_{m}=0.25,0.5,0.75$. When correcting the alpha spending functions at each analysis (solid line), we used a per-test two-sided nominal level of $p^{*}=0.035$ to calculate the four boundaries and alpha spent at each analysis time. In contrast, when correcting the alpha spending functions only at the final analysis (dashed line), we constructed a worst-case example of overestimating the correlation: the first three interim boundaries are set using $p^{*}=0.05$ as if the co-primary tests were perfectly correlated and required no multiplicity adjustment, and only the final boundary is re-calibrated so that the overall per-test nominal level is $p^{*}\approx 0.035$ based on the observed correlation. The two plots showed little difference between the boundaries as well as the alpha to be spent at each test.

However, other types of boundaries may spend more type I error earlier in the trial, resulting in a greater impact of waiting until the final analysis to correct the $p^{*}$. For example, when using Pocock boundaries, the type I error is equally spent across all M analyses. The inherent uncertainty in key parameters when designing the trial may cause over-spending of the type I error prior to the final analysis, especially when M is large. Fig. 1 panels c and d show an example of overspending when using a Pocock-type alpha spending function. When using a $p^{*}$ level of 0.05 to calculate the first three interim analyses, the alpha spent was already above 0.035 for three interim analyses, leaving no alpha to be spent at the final analysis. In these circumstances, one may consider using the observed data to adjust the alpha spending functions at each interim analysis and reducing the number of early planned interim analyses.

## 4 Application to the OTAC trial

This section presents the steps of applying the correlation correction to the OTAC trial based on the original assumptions described in Section 2.

To obtain initial estimates of the standard errors for both test statistics, we calculated the proportion of participants in each ordinal category by treatment group and stratum using the assumptions. The results are presented in Table 1. We then constructed an exemplary dataset with 20 rows representing each combination of the 5 ordinal categories, 2 strata, and 2 treatments, with a column showing the expected proportion of patients in each category. A proportional odds model was fit in each stratum, respectively. For each stratum, we first obtained the model-based variance of the treatment effect under an effective sample size of 1 by using the observed proportions in each outcome category; this yielded a standardized standard error corresponding to n=1, which we then rescaled to the actual stratum sample size. Supplementary Table S3 shows the sample construction of exemplary dataset for the OTAC Trial. The correlation between two test statistics was then derived from Eq. (4).

The mean and variance–covariance matrix for the bivariate normal distribution of the two test statistics under the null hypothesis was derived as described in Section 3.1.1. We iterated through values for $p^{*}$ from 0.025 to 0.05 in increments of 0.0001 to obtain the probability of rejecting either null hypothesis given both are true $P\left(\left(\left|Z\right|\geq c\right)\cup \left(\left|Z_{1}\right|\geq c\right)|H_{0},H_{0,1}\right)$. The maximum $p^{*}$ that maintained an overall type I error rate below 0.05 was 0.0347; this was specified as our nominal p-value at the beginning of the trial. The nominal p-value will be recalculated at the end of the actual trial using the observed data. Proceeding with the nominal p-value of 0.0347, we identified the smallest sample size that yielded a power of more than 80% under the alternative hypotheses to be 780 participants. After accounting for loss to follow-up, the total sample size was inflated to 820.

**Table 1:** Assumed distribution of ordinal outcomes for different treatment groups and strata in the OTAC trial.

	Stratum 1			Stratum 2	
	Ordinal	Proportion		Ordinal	Proportion
Placebo	1	0.20		1	0.30
	2	0.40		2	0.42
	3	0.25		3	0.19
	4	0.12		4	0.08
	5	0.03		5	0.02

Treatment	1	0.27		1	0.34
	2	0.42		2	0.42
	3	0.20		3	0.17
	4	0.09		4	0.06
	5	0.02		5	0.01

## 5 Simulation

### 5.1 Simulation methods

We simulated trials with two strata, evaluating co-primary hypotheses for the full cohort and for Stratum 1. The two null hypotheses, denoted by $H_{0}$ and $H_{01}$, state no treatment effect for the full cohort and stratum 1, respectively. We consider the trial a “success” if either of the null hypothesis is rejected and define the type I error as the rate at which either null hypothesis is rejected given that both are true, using two-sided tests.

We compared the proposed correlation correction with Bonferroni correction in terms of the type I error rate, power, and expected sample size (ESS) (when applicable) under various conditions. We used a Bonferroni correction as the benchmark, rather than Holm’s method or a gatekeeping approach to evaluate our approach for the following reasons. Although Holm’s method is commonly used, it yields identical results to Bonferroni in terms of power and type I error rate for this scenario, since the family-wise error rate depends solely on the minimum p-value compared with the overall type I error rate divided by the number of tests. Gate-keeping procedures were not considered because they require a predefined hierarchical ordering of hypotheses, which conflicts with our objective of concurrently evaluating endpoints of equal clinical priority.

The proportion of patients in stratum 1 P(S1) was varied from 0.9 to 0.5. For the distribution of ordinal outcome in the stratum 1 placebo group, P(S1placebo), we examined two sets of proportions for each category: (0.2,0.4, 0.25,0.12,0.03) and (0.3,0.5,0.12,0.03,0.02); the first set of proportions is aligned with our hypotheses in the OTAC trial, the second set is the scenario when we observe fewer severe outcomes. Among these 10 conditions, the one aligned with the sample size assumptions in the OTAC trial with a sample size of 780 estimated to maintain 80% power and a type I error rate of 0.05 was used as the “fixed” sample size to assess the potential effect of deviations from the original assumption without interim analysis; this sample size was then inflated to 792 due to three planned interim analyses. In all cases, we set the summary odds ratio (OR) to be 1.72 comparing outcomes in the placebo group in stratum 2 to the placebo group in stratum 1 under the null hypothesis; the ORs for the treatments in stratum 1 and stratum 2 compared to placebo were set to be 1.5 and 1.2, respectively, under the alternative hypothesis.

In the first set of simulations, we evaluated the type I error rate control of the proposed method without interim analyses across various sample size assumptions. Data were first generated with the sample size and the nominal p-value estimated to maintain a type I error rate of 0.05 and power of 0.8 under each scenario. The correlation of the two test statistics, the nominal p-value, and the sample size were estimated based on the trial assumptions. The “actual” type I error rate and power were estimated from the simulation. We also compared the necessary sample size to maintain a power of 0.8 between the proposed approach and when using Bonferroni correction. We then fixed the sample size of 780 and simulated the type I error rate and power under deviations from these sample size assumptions. The nominal p-value for the Bonferroni correction was set at 0.025 and was determined for the correlation correction based on the observed standard errors for each simulation iteration.

A second set of simulations was then carried out with 3 interim analyses at t=25%,50%, and 75% of enrollment. We first fixed the power, varied the sample size as in the first part, and obtained the ESS with interim monitoring through simulation. The boundaries for each interim analysis were obtained from a Lan-Demets O’Brien Fleming-like alpha spending function using the nominal p-value determined based on the assumptions used to calculate the sample size. In the final analysis, we adjusted the alpha spending functions using the nominal p-value estimated from the full data. However, because the data were generated in accordance with the sample size assumptions, we would not expect large differences between the assumed and estimated correlation between the test statistics at the end of the trial and thus for adjustment of the boundaries to meaningfully impact the results. We then fixed the sample size to simulate the type I error rate and power with both methods under different deviations from the sample size assumptions. We compared the results by only adjusting the alpha spending functions at the final analysis (using the nominal p-value for the assumed scenario to calculate the first three boundaries), and adjusting at each analysis using the nominal p-value obtained from the current simulated data. Similar simulations for a time-to-event outcome are detailed in the Supplementary Material.

All simulations were carried out using R version 4.3.1. Proportional odds regression models were carried out to evaluate the treatment effect using the “MASS” package [41]; the nominal p-value was obtained using the “mvtnorm” package [42]; boundaries for interim analyses were obtained using the “gsDesign” package [43]. Each scenario included 10,000 iterations.

### 5.2 Simulation results

#### 5.2.1 Without interim monitoring

Table 2 presents simulation results for the ordinal outcome scenarios without interim analysis. As the proportion of patients in each stratum became more balanced, the correlation decreased and the required sample size under the correlation correction increased. When P(S1placebo) was assumed to be (0.2,0.4,0.25,0.12,0.03), as the P(S1) decreased from 0.9 to 0.5, the estimated correlation between the two test statistics decreased from 0.949 to 0.709. The sample size needed to maintain the same power for both methods correspondingly increased from 705 to 1141. The Bonferroni correction was underpowered compared to correlation correction and had a type I error rate lower than the specified rate of 0.05. The correlation correction method thus required a significantly smaller sample size to achieve an 80% power compared to the Bonferroni correction, especially in situations with larger correlations. As shown in the last column of Table 2, when P(S1) was 90%, the correlation correction saved 11.1% of sample size compared to Bonferroni correction, and 4.5% when P(S1) was 50%. When P(S1placebo) was assumed to be (0.3,0.5,0.15,0.03,0.02), we observed similar patterns, though a larger sample size was necessary to achieve the same power and type I error.

When fixing the sample size and P(S1placebo), power was greater when the correlation between test statistics was higher. Though power differed from the targeted 80% when the sample size assumptions did not hold, the correlation correction method consistently controlled the type I error at approximately 0.05. Compared to the Bonferroni correction, the correlation correction provided greater power in all circumstances, with the difference in power for the two methods ranging from 0.023 to 0.046, and showed a larger difference when the correlation between test statistics was higher. Similar results were found for the time-to-event outcome (Supplementary Tables S1 & S2).

**Table 2:**
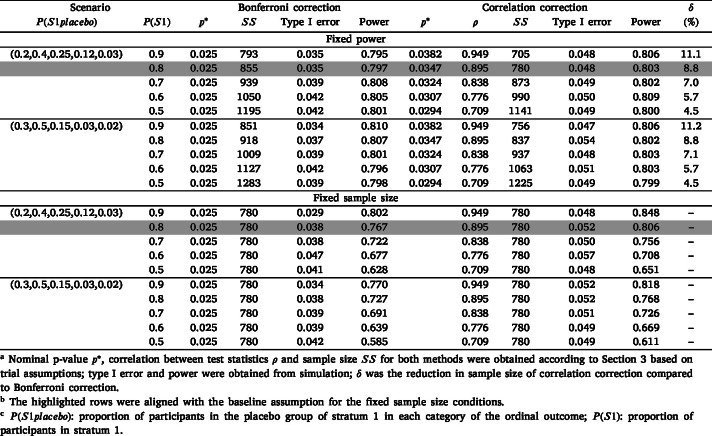
Simulation results without interim analysis for ordinal outcome.

#### 5.2.2 With interim monitoring

Table 3 presents the simulation results for ordinal outcomes with three interim analyses.

When adding three interim analyses, the expected sample size (ESS) under the alternative hypothesis was reduced compared to the planned sample size due to the early termination of trials, with the ESS when using the correlation correction being smaller compared to the Bonferroni correction. Taking the conditions where P(S1placebo) was (0.2,0.4,0.25,0.12,0.03) for example, the reduction in ESS for the correlation correction method compared to the Bonferroni correction ranged from 11.7% when P(S1) was 90% to 4.8% when P(S1) was 50%.

**Table 3:**
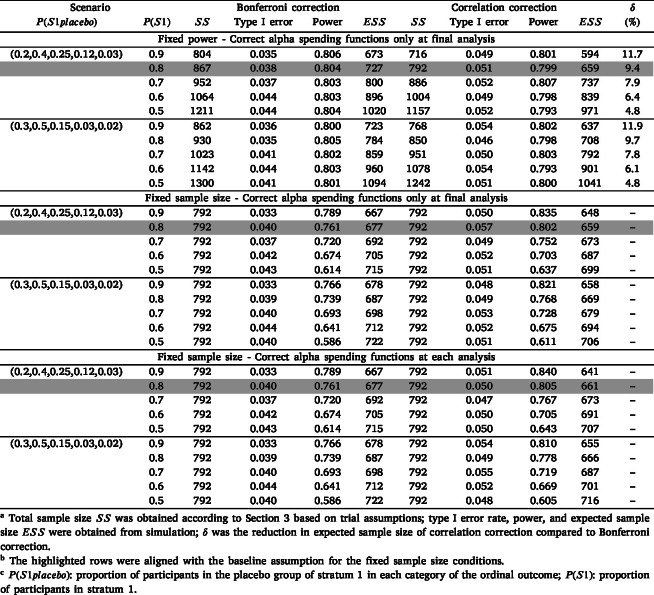
Simulation results with interim analysis for ordinal outcome.

We also hypothesized that the power and ESS would differ when adjusting the interim monitoring boundaries only at the final analysis compared to adjusting at each analysis to account for differences in the nominal p-value approximated at the beginning of the trial and the value ultimately used based on the observed data. This was because the first three boundaries were determined based on a nominal p-value of 0.0347 (the nominal p-value aligned with the original sample size assumptions) when only adjusting at the final analysis. We assumed that when only adjusting the boundaries at the final analysis, if the pre-specified correlation was larger than the true correlation, more type I error would be spent in the first three interim analyses than intended, leaving less type I error for the final analysis.

We observed that adjusting the boundaries only at the final analysis resulted in a slightly smaller ESS when the assumed correlation was larger than the true correlation, and slightly larger when the assumed correlation was smaller than the true correlation. No clear trend with respect to power was observed. For all sets of simulations, we observed similar trends for the time-to-event outcome.

## 6 Discussion

In the OTAC trial, type I error for the co-primary tests of the treatment effect in the overall population and in stratum 1 will be controlled by lowering the nominal p-value based on the estimated correlation between the two tests. We assessed type I error and power using this method by simulating the results for both an ordinal and time-to-event outcome under varying scenarios, both without and with interim analyses. The performance of the proposed correlation correction is robust to deviations from sample size assumptions, consistently maintaining a desired type I error rate of approximately 0.05 under all simulation settings even when the sample size was inadequate to maintain the specified power. As expected, this also reduces the required sample size compared to a Bonferroni correction, especially when the correlation between two test statistics is large.

The correlation between tests is similar among different treatment effects and baseline conditions given the same proportion of patients in important subgroups. Correcting the alpha spending functions for the co-primary tests based on the estimated correlation at each interim analysis reduces the ESS when the correlation between tests is higher than initially anticipated (i.e. more participants in stratum 1) by allowing more type I error to be spent earlier, but leaving less for the final analysis. When the correlation is lower than anticipated, correcting the alpha spending functions at each analysis results in a slightly larger ESS compared to correcting the alpha spending functions only at the final analysis. This is because less type I error is allocated to interim analyses once the nominal p-value is adjusted. This trend was demonstrated in Section 5.2.2 through the simulations with a fixed total sample size.

While in all cases we divided the type I error equally for each hypothesis, it is straightforward to allocate more or less type I error rate for some of the hypothesis tests by adjusting the critical values in Eq. (6). In this paper, we did not specifically discuss the methods for correcting the boundaries in conjunction with a sample size re-estimation; this could be an important topic for future work. The method described also has some additional limitations. First, this assumes that the test statistics follow a multivariate normal distribution and thus may not be applicable to all primary outcomes or analyses when the sample size is too small to rely on asymptotic theory. Second, when the correlation was smaller than 0.7 (e.g. when P(S1) was less than 50% in the ordinal outcome scenarios), the reduction in necessary sample size was less than 5% compared to the Bonferroni method. In cases where the correlation between test statistics is low, there may be minimal benefit over the Bonferroni correction.

Although our simulations focused on an O’Brien–Fleming-like spending function, the proposed correlation correction can be applied with other spending functions such as those resembling a Pocock boundary. Because a Pocock boundary allocates more type I error at earlier looks, mis-specification of the correlation can lead to more pronounced early overspending. In such settings, updating the nominal p-value at each interim analysis rather than relying solely on a final-analysis adjustment is expected to be particularly important to preserve type I error control. By contrast, with an O’Brien–Fleming–like spending function that spends very little early, updating the correlation-adjusted alpha function only at the final look typically has a negligible impact on power while maintaining type I error control.

From a design perspective, our proposed method is most useful when there are only a few co-primary strata (e.g., the overall population plus one pre-specified subgroup). Adding more disjoint strata splits the sample and weakens correlations between test statistics, reducing the efficiency gain over a Bonferroni correction.

In the OTAC protocol, demonstration of efficacy in either the overall population or in Stratum 1 was specified as a stopping criterion for further enrollment. Because Stratum 1 was expected to constitute approximately 80% of participants and the co-primary hypotheses are defined for Stratum 1 and for the overall cohort, stopping only in Stratum 1 while continuing enrollment in Stratum 2 would effectively reveal that the overall effect is likely favorable, creating substantial risk of operational unblinding and complicating interpretation of any subsequent data. For this reason, we do not recommend designs that stop a single stratum while continuing others once efficacy has been declared. In alternative designs with multiple subgroups of interest, one could in principle allow enrollment to continue in the remaining subgroup(s) after rejecting the null hypothesis in a given subgroup, but the same concerns about partial unblinding and interpretability remain. If such a strategy were nevertheless adopted, the post-rejection monitoring problem would simply involve a reduced set of primary hypotheses, and our proposed method could be applied by updating at each subsequent analysis, (1) the joint correlation structure for the remaining test statistics and (2) the corresponding alpha-spending function.

In summary, the correlation correction method controlled the type I error at the desired rate under all simulation scenarios. It also provides the most benefit in sample size reduction compared to Bonferroni correction when the correlation between the test statistics is large (up to 12% in our simulation setting when P(S1) was 90%). This robust approach provides a feasible method for controlling the type I error rate and leveraging the correlation between multiple tests to increase power. This case study can serve as a useful reference for future studies involving similar endpoint structures, especially when subgroup inference is of clinical or regulatory importance.

## CRediT authorship contribution statement

**Jiayi Hu:** Writing – review & editing, Writing – original draft, Software, Methodology, Conceptualization. **Abdel G. Babiker:** Writing – review & editing, Project administration, Methodology, Conceptualization. **Cavan S. Reilly:** Writing – review & editing, Project administration, Methodology, Funding acquisition, Conceptualization. **Jason V. Baker:** Writing – review & editing, Project administration. **Lianne K. Siegel:** Writing – review & editing, Writing – original draft, Supervision, Software, Project administration, Methodology, Conceptualization.

## Declaration of competing interest

The authors declare that they have no known competing financial interests or personal relationships that could have appeared to influence the work reported in this paper.

## Data Availability

No data was used for the research described in the article.

## Appendix A Supplementary data

MMC S1 . MMC S1
