# Supplementary material for: Type I error control and interim monitoring for co-primary hypotheses involving a subgroup in the Outpatient Treatment with Anti-Coronavirus Immunoglobulin (OTAC) trial
Source: Contemp Clin Trials Commun. 2025 Dec 24;49:101592. doi: 10.1016/j.conctc.2025.101592 (PMC12809130; doi:10.1016/j.conctc.2025.101592)
Supplement: MMC S1 — . [file mmc1.pdf]

Supplementary Materials for Type I Error Control and Interim Monitoring for Co-Primary Hypotheses Involving a Subgroup in the Outpatient Treatment with Anti-Coronavirus Immunoglobulin (OTAC) Trial by

Jiayi Hu[1], Abdel G. Babiker[2], Cavan S. Reilly[1], Jason V. Baker[3,4], and Lianne K. Siegel[1],
for the STRIVE OTAC Study Group
[1]Division of Biostatistics and Health Data Science, University of Minnesota, Minnesota, U.S.A.
[2]Medical Research Council Clinical Trials Unit at UCL, University College London, London, U.K.
[3]Division of Infectious Diseases and International Medicine, University of Minnesota, Minnesota, U.S.A.
[4]Division of Infectious Diseases, Hennepin Healthcare, Minneapolis, Minnesota, U.S.A.

November 30, 2025

1 R code for determining the correlation between two test statistics in the OTAC trial.

```
library(ordinal)
library(Hmisc)

getp1 <- function(p,theta){
  k <- length(p)
  qc <- c()
  qe <- c()
  qei <- c()
  pe <- c()
  for (i in 1:k){
    qc <- append(qc,sum(p[1:i]))
    qei <- qc[i] / (qc[i] + (1-qc[i]) * exp(-theta))
    qe <- append(qe, qei)
  }
  pe[1] <- qe[1]
  for (i in 2:k) {
    pe[i] <- qe[i] - qe[i-1]
  }
  pe
}

pPlcbS1<- c(0.2,0.4,0.25,0.12,0.03)
pTrtS1 <- getp1(pPlcbS1, log(1.5))
```

```
pPlcbS2 <- getp1(pPlcbS1,log(1.72))
pTrtS2 <- getp1(pPlcbS2,log(1.2))

dat1 <- data.frame(y=factor(rep(1:5,4)),z=rep(c(rep(0,5),rep(1,5)),2),
    s=c(rep(1,10),rep(0,10)))
prob1 <- c(c(pPlcbS1,pTrtS1)*0.8, c(pPlcbS2,pTrtS2)*0.2) * 0.5

model_s1 <- clm(y~z, data=dat1, weights=prob1, subset = s==1)
se1 <- summary(model_s1)\$coef[5,2]
model_s2 <- clm(y~z, data=dat1, weights=prob1, subset = s==0)
se2 <- summary(model_s2)\$coef[5,2]
rho1 <- sqrt(1 / (1 + se1^2/se2^2))
```

2 Simulation for Time-to-event Outcomes

2.1 Simulation Methods

We repeated the simulation conditions (as discussed in the simulation methods of the main paper) using a time-to-event outcome (e.g. time from randomization to death) with data simulated under a proportional hazards model. We assumed the survival times of patients in stratum 1 and stratum 2 followed exponential distributions with different 28-day survival rates and different hazard ratios comparing the two treatment groups. The proportion of patients in stratum 1 varied from 0.9 to 0.5, and the 28-day survival probability in the placebo groups for the two strata $S(28placebo)$ was set either to be $(0.2, 0.5)$ or $(0.4, 0.6)$; the hazard ratios (HR) were set at 0.7 and 0.85 comparing the treatment group versus placebo group in stratum 1 and stratum 2, respectively, under the alternative hypothesis. These data were generated using the "simsurv" R package [1].

Simulations both with and without interim analyses were conducted under fixed power and fixed sample size designs, respectively, as previously described in the main paper. In the set of 10 conditions, the scenario where the proportion of patients in stratum 1 $P(S1)$ was 0.8 and the $S(28placebo)$ was $(0.4, 0.6)$ was chosen as the baseline assumption for the fixed sample size conditions. In this case, to achieve 80% power and a type I error of 0.05, the required sample size was determined to be 572 without interim analysis and was inflated to 582 after accounting for three planned interim analyses. Cox proportional hazards regression models were fitted to evaluate the treatment effect.

2.2 Simulation Results

Table S1 presents the simulation results for time-to-event outcomes without interim analyses. For the results under the fixed power settings, the type I error rate was steadily controlled at about 0.05; the sample size required to maintain a type I error of 0.05 and power of 80% increased with the proportion of patients enrolled in stratum 1 in both methods, and the correlation correction had a significantly decreased sample size compared to Bonferroni correction.

The total sample size was then fixed at 572. The results were similar for the fixed sample size settings between the time-to-event and ordinal outcomes. The type I error was steadily controlled at approximately 0.05, with higher power for the correlation correction compared to the Bonferroni Correction in all conditions. The power of the trial decreased as the proportion of patients in stratum 1 decreased.

Table S2 provides the simulation results for the time-to-event outcome with three interim analyses. The results we obtained were similar to the results for ordinal outcomes. Under the conditions

with fixed power, the correlation correction consistently controlled the type I error at 0.05, with a reduction in expected sample size (ESS) compared to the Bonferroni correction while maintaining 80% power (from 12.1% to 5.5% as shown in Table S2).

Under the conditions with a fixed sample size, the power decreased as the $P(S1)$ decreased. Similar to what we observed in the ordinal outcomes: When we adjusted the boundaries only in the final analysis, if the correlation we assumed at the beginning of the trial was larger than the true correlation, the ESS would be slightly smaller and the power would be slightly smaller than if we adjusted the boundaries in each analysis; if we assumed a correlation that was smaller than the true correlation, we would observe a slightly larger ESS and a slightly larger power compared to adjust the boundary at each analysis.

3 Algorithm for Correlation-based Alpha Allocation and Boundary Update

Algorithm S1 Correlation-based alpha allocation and boundary update

Require: Target family-wise type I error α; pre-specified spending function $\alpha_*(t)$; planned design parameters; total number of analyses M; blinded realized stratum counts / information used to estimate the current correlation.

for $m = 1, \ldots, M$ **do**

  On a grid of candidate two-sided nominal p-values, derive p^*_{new} satisfying Equation (6) using the *observed* sample information at look m.

  Update the critical value at look m, c_m, based on Equation (7) and p^*_{new}.

  At look m, if the corresponding $|Z(t_m)|$ or $|Z_i(t_m)|$ exceeds c_m, reject H_0 and/or $H_{0,i}$, declare trial efficacy, and stop further looks. Otherwise, continue to the next planned analysis.

  if $m < M$ **and** no rejection has occurred at look m **then**

    Update the spending function $\alpha'_*(t)$ for $t > t_m$ using p^*_{new} and $\alpha_*(t)$ as in Equation (8), so that the overall family-wise type I error rate remains controlled at α.

  end if

end for

4 Supplementary Tables

Table S1: Simulation Results without Interim Analysis for Time-to-Event Outcome

**Scenario**		**Bonferroni Correction**				**Correlation Correction**					δ
$S(28placebo)$	$P(S1)$	p^*	SS	Type I error	power	p^*	ρ	SS	Type I error	power	(%)
					**Fixed Power**						
(0.2,0.5)	0.9	0.025	423	0.033	0.800	0.0400	0.966	372	0.051	0.800	12.1
	0.8	0.025	460	0.039	0.794	0.0366	0.928	414	0.049	0.807	10.0
	0.7	0.025	510	0.035	0.804	0.0343	0.885	466	0.048	0.800	8.6
	0.6	0.025	575	0.038	0.802	0.0324	0.837	534	0.048	0.801	7.1
	0.5	0.025	662	0.042	0.799	0.0308	0.780	624	0.048	0.805	5.7
(0.4,0.6)	0.9	0.025	584	0.030	0.794	0.0396	0.963	514	0.050	0.802	12.0
	0.8	0.025	634	0.037	0.809	0.0362	0.922	572	0.047	0.804	9.8
	0.7	0.025	700	0.037	0.806	0.0338	0.876	644	0.052	0.802	8.0
	0.6	0.025	788	0.042	0.804	0.0320	0.824	735	0.049	0.798	6.7
	0.5	0.025	906	0.041	0.799	0.0305	0.765	856	0.050	0.806	5.5
					**Fixed Sample Size**						
(0.2,0.5)	0.9	0.025	572	0.032	0.909		0.966	572	0.051	0.935	-
	0.8	0.025	572	0.035	0.884		0.928	572	0.050	0.912	-
	0.7	0.025	572	0.037	0.848		0.885	572	0.052	0.878	-
	0.6	0.025	572	0.041	0.788		0.837	572	0.050	0.817	-
	0.5	0.025	572	0.043	0.745		0.780	572	0.052	0.770	-
(0.4,0.6)	0.9	0.025	572	0.034	0.792		0.963	572	0.052	0.843	-
	0.8	0.025	572	0.031	0.757		0.922	572	0.048	0.806	-
	0.7	0.025	572	0.036	0.714		0.876	572	0.051	0.756	-
	0.6	0.025	572	0.037	0.651		0.824	572	0.048	0.686	-
	0.5	0.025	572	0.039	0.598		0.765	572	0.048	0.629	-

[a] Nominal p-value p^*, correlation between test statistics ρ and sample size SS for both methods were obtained based on trial assumptions in the subsection *Simulation Methods*; type I error and power were obtained from simulation; δ was the reduction in sample size of correlation correction compared to Bonferroni correction.

[b] The highlighted rows were aligned with the baseline assumption for the fixed sample size conditions.

[c] $P(28placebo)$: 28-day survival probability in the placebo group;
$P(S1)$: proportion of participants in stratum 1.

Table S2: Simulation Results with Interim Analyses for Time-to-Event Outcome

**Scenario**		**Bonferroni Correction**				**Correlation Correction**				δ
$S(28placebo)$	$P(S1)$	SS	type I error	power	ESS	SS	type I error	power	ESS	(%)
**Fixed Power - Correct boundary only at final analysis**										
(0.2,0.5)	0.9	431	0.034	0.807	362	379	0.049	0.803	315	13.0
	0.8	469	0.033	0.799	396	422	0.047	0.804	352	11.1
	0.7	520	0.040	0.799	438	475	0.045	0.797	398	9.1
	0.6	586	0.040	0.799	493	544	0.051	0.801	455	7.7
	0.5	675	0.040	0.802	569	636	0.044	0.807	533	6.3
(0.4,0.6)	0.9	595	0.030	0.801	501	524	0.047	0.802	435	13.2
	0.8	646	0.037	0.803	543	583	0.054	0.805	485	10.7
	0.7	713	0.034	0.801	600	656	0.048	0.803	545	9.2
	0.6	803	0.039	0.803	677	749	0.048	0.798	626	7.5
	0.5	923	0.039	0.806	774	872	0.051	0.806	732	5.4
**Fixed Sample Size - Correct boundary only at final analysis**										
(0.2,0.5)	0.9	583	0.032	0.910	453	583	0.050	0.938	436	-
	0.8	583	0.036	0.885	465	583	0.053	0.913	448	-
	0.7	583	0.037	0.850	477	583	0.050	0.876	460	-
	0.6	583	0.037	0.801	492	583	0.051	0.824	474	-
	0.5	583	0.037	0.732	506	583	0.047	0.754	490	-
(0.4,0.6)	0.9	583	0.030	0.802	493	583	0.047	0.850	476	-
	0.8	583	0.035	0.758	502	583	0.049	0.803	486	-
	0.7	583	0.035	0.713	510	583	0.049	0.751	495	-
	0.6	583	0.037	0.655	522	583	0.047	0.687	507	-
	0.5	583	0.040	0.588	532	583	0.050	0.614	519	-
**Fixed Sample Size - Correct boundary at each analysis**										
(0.2,0.5)	0.9	583	0.032	0.910	453	583	0.050	0.937	430	-
	0.8	583	0.036	0.885	465	583	0.053	0.913	447	-
	0.7	583	0.037	0.850	477	583	0.051	0.877	462	-
	0.6	583	0.037	0.801	492	583	0.051	0.826	480	-
	0.5	583	0.037	0.732	506	583	0.047	0.757	497	-
(0.4,0.6)	0.9	583	0.030	0.802	493	583	0.048	0.849	471	-
	0.8	583	0.035	0.758	502	583	0.048	0.803	486	-
	0.7	583	0.035	0.713	510	583	0.050	0.752	498	-
	0.6	583	0.037	0.655	522	583	0.048	0.690	512	-
	0.5	583	0.040	0.588	532	583	0.050	0.618	525	-

[a] Total sample size SS was obtained based on trial assumptions described in the subsection $SimulationMethods$; type I error rate, power, and expected sample size ESS were obtained from simulation; δ was the reduction in expected sample size of correlation correction compared to Bonferroni correction.

[b] The highlighted rows were aligned with the baseline assumption for the fixed sample size conditions.

[c] $P(28placebo)$: 28-day survival probability in the placebo group;
$P(S1)$: proportion of participants in stratum 1.

Table S3: Sample Construction of Exemplary Dataset for the OTAC Trial

Stratum	Treatment	Ordinal	Proportion
1	0	1	0.080
1	0	2	0.160
1	0	3	0.100
1	0	4	0.048
1	0	5	0.012
1	1	1	0.109
1	1	2	0.168
1	1	3	0.081
1	1	4	0.034
1	1	5	0.008
2	0	1	0.030
2	0	2	0.042
2	0	3	0.019
2	0	4	0.008
2	0	5	0.002
2	1	1	0.034
2	1	2	0.042
2	1	3	0.017
2	1	4	0.006
2	1	5	0.001

[a] The placebo group is coded as 0 and the treatment group is coded as 1 in the Treatment column.

Table S4: Theoretical Correlation ρ, Nominal P-value p^*, and Sample Size SS maintaining alpha of 0.05 and power of 80% under Different Treatment Effects and $P(S1)$

Treatment Effect	$P(S1)$	ρ	p^*	SS
HR(0.8,0.9)	0.9	0.967	0.0400	914
	0.8	0.929	0.0367	1016
	0.7	0.887	0.0343	1144
	0.6	0.839	0.0325	1307
	0.5	0.782	0.0309	1524
HR(0.7,0.85)	0.9	0.966	0.0400	372
	0.8	0.928	0.0366	414
	0.7	0.885	0.0343	466
	0.6	0.837	0.0324	534
	0.5	0.780	0.0308	624
HR(0.6,0.8)	0.9	0.965	0.0399	191
	0.8	0.927	0.0365	213
	0.7	0.883	0.0342	240
	0.6	0.834	0.0323	275
	0.5	0.776	0.0307	322
OR(1.35,1.1)	0.9	0.949	0.0383	1292
	0.8	0.895	0.0347	1453
	0.7	0.838	0.0324	1653
	0.6	0.776	0.0307	1912
	0.5	0.709	0.0294	2260
OR(1.5,1.2)	0.9	0.949	0.0382	705
	0.8	0.895	0.0347	780
	0.7	0.838	0.0324	873
	0.6	0.776	0.0307	990
	0.5	0.709	0.0294	1141
OR(1.65,1.3)	0.9	0.949	0.0382	462
	0.8	0.895	0.0347	507
	0.7	0.837	0.0324	561
	0.6	0.775	0.0307	628
	0.5	0.708	0.0294	712

[a] HR(num1,num2) (or OR(num1,num2)) refers to the hazard ratio (or odds ratio) for the treatment group compared to the placebo group in stratum1 and stratum2, respectively.

References

[1] Brilleman, S.L., Wolfe, R., Moreno-Betancur, M., and Crowther, M.J. (2021). Simulating survival data using the simsurv R package. *Journal of Statistical Software*, **97**, 1–27.
